# Comparisons of molecular diversity indices, selective sweeps and population structure of African rice with its wild progenitor and Asian rice

**DOI:** 10.1007/s00122-018-3268-2

**Published:** 2018-12-21

**Authors:** Marie Noelle Ndjiondjop, Nikolaos Alachiotis, Pavlos Pavlidis, Alphonse Goungoulou, Sèdjro Bienvenu Kpeki, Dule Zhao, Kassa Semagn

**Affiliations:** 1 M’bé Research Station, Africa Rice Center (AfricaRice), 01 B.P. 2551, Bouaké 01, Côte d’Ivoire; 2 Institute of Computer Science, Foundation for Research and Technology-Hellas, Nikolaou Plastira 100, 70013 Heraklion, Crete Greece

## Abstract

***Key message*:**

**The extent of molecular diversity parameters across three rice species was compared using large germplasm collection genotyped with genomewide SNPs and SNPs that fell within selective sweep regions.**

**Abstract:**

Previous studies conducted on limited number of accessions have reported very low genetic variation in African rice (*Oryza glaberrima* Steud.) as compared to its wild progenitor (*O. barthii* A. Chev.) and to Asian rice (*O. sativa* L.). Here, we characterized a large collection of African rice and compared its molecular diversity indices and population structure with the two other species using genomewide single nucleotide polymorphisms (SNPs) and SNPs that mapped within selective sweeps. A total of 3245 samples representing African rice (2358), Asian rice (772) and *O. barthii* (115) were genotyped with 26,073 physically mapped SNPs. Using all SNPs, the level of marker polymorphism, average genetic distance and nucleotide diversity in African rice accounted for 59.1%, 63.2% and 37.1% of that of *O. barthii*, respectively. SNP polymorphism and overall nucleotide diversity of the African rice accounted for 20.1–32.1 and 16.3–37.3% of that of the Asian rice, respectively. We identified 780 SNPs that fell within 37 candidate selective sweeps in African rice, which were distributed across all 12 rice chromosomes. Nucleotide diversity of the African rice estimated from the 780 SNPs was 8.3 × 10^−4^, which is not only 20-fold smaller than the value estimated from all genomewide SNPs (*π* = 1.6 × 10^−2^), but also accounted for just 4.1%, 0.9% and 2.1% of that of *O. barthii*, lowland Asian rice and upland Asian rice, respectively. The genotype data generated for a large collection of rice accessions conserved at the AfricaRice genebank will be highly useful for the global rice community and promote germplasm use.

**Electronic supplementary material:**

The online version of this article (10.1007/s00122-018-3268-2) contains supplementary material, which is available to authorized users.

## Introduction

Rice (*Oryza* L.) is one of the top three food crops in the world, ranked second in total area harvested and production per hectare and third in total global production after maize and wheat (http://www.fao.org/faostat/en/#data; accessed in Dec. 2018). The genus *Oryza* consists of 27 species and 11 genome types (Stein et al. 2018). Eight species encompassing five genomes are found on the African continent, which include the AA (*Oryza barthii* A. Chev., *O. glaberrima* Steud., *O. sativa* L., and *Oryza longistaminata* Chev. and Röhr), BB (*O. punctata*), CC (*O. eichingeri*), FF (*O. brachyantha*) and BBCC (*O. schweinfurthiana*) genome species (Vaughan et al. 2003). All these species are conserved at the AfricaRice genebank, each with a varying number of accessions ranging from few to several thousands. Of all the ~ 22,000 registered rice samples conserved at the AfricaRice genebank, about 14% belong to African rice (*O. glaberrima*), 85% to Asian rice (*O. sativa*) and 1% of the other wild species.

African and Asian rice have been shown to be independently domesticated from the wild species of *O. barthii* in Africa and *O. rufipogon* in Asia, respectively (Wang et al. 2014). Recent studies of the genetic variation, relatedness and population structure of 2179 African rice accessions indicated that overall level of polymorphism and genetic distance between pairs of accessions observed across this large number of samples were very low (Ndjiondjop et al. 2017). Other studies compared the extent of genetic variation of African rice using whole genome sequencing and targeted sequencing of a few genes that have undergone selection (Li et al. 2011; Nabholz et al. 2014; Wang et al. 2014; Meyer et al. 2016; Win et al. 2017; Cubry et al. 2018; Lv et al. 2018) and reported very low genetic variation in African rice as compared to Asian rice and to its wild progenitor *O. barthii*. Results of the gene-based sequencing studies suggest a strong domestication bottleneck, while those of genomewide studies suggested the role of both genetic bottleneck and selective sweeps as the main factors for the lower genetic diversity observed in the cultivated African rice compared to its wild progenitor (Vaughan et al. 2008). However, all previous studies that compared the extent of nucleotide diversity and effect of selective sweeps in African rice and *O. barthii* were based on small number of accessions, ranging from 9 to 163 and from 10 to 88 samples, respectively. Nucleotide diversity in such a small sample may be depressed more based on the sample size itself, than on evolutionary forces acting on the entire species, which forms one of the bases of this study.

Selection (selective) sweeps, which refer to a reduction in nucleotide diversity near advantageous mutations in useful genes, leave distinct signatures in genomes; this enables the detection of loci that have undergone positive selection (Peter et al. 2012; Chen et al. 2016; Alachiotis and Pavlidis 2018). Positive selection increases the frequency of a beneficial allele within a population and may even lead to fixation. While this process increases fitness of the individuals carrying the beneficial allele, it reduces overall genetic diversity in a population or species until recombination and mutation introduce new alleles in these selected regions (Olsen et al. 2006; Vitti et al. 2013; Alachiotis and Pavlidis 2016). For some time, however, the selected alleles are present at high frequency, while the newly introduced alleles (or residual, less beneficial alleles) are found at low frequency, and may remain so unless they are neutral or beneficial in effect. Using data from small number of samples, several studies reported a reduction in genetic diversity in domesticated (cultivated) crops as compared to their wild progenitors (Reif et al. 2005; Wright et al. 2005; Gore et al. 2009; Lam et al. 2010; Ding et al. 2011; Huang et al. 2012; Yuan et al. 2017), but those results may also be biased by samples sizes. Thus, assessment of the level of nucleotide diversity in large number of individuals using both genomewide markers and a subset of markers that physically map within selective sweep regions would provide confidence, which forms another basis of this study.

A wide range of statistical methods have been used to identify traces of intra-species selective sweeps by detecting regions of reduced genetic variation, which have undergone a selective sweep (Crisci et al. 2013; Alachiotis and Pavlidis 2016; Pavlidis and Alachiotis 2017). Using simulated data, Crisci and colleagues evaluated SweepFinder (Nielsen et al. 2005), SweeD (Pavlidis et al. 2013), OmegaPlus (Alachiotis et al. 2012) and iHS (Voight et al. 2006) in terms of efficiency on type I and II errors, effect of population structure and size and genome coverage. Overall, OmegaPlus performed better than the other three methods. In the present study, we used DArTseq genotyping of 3245 samples representing African rice, Asian rice and *O. barthii* to (1) identify genomic regions that have undergone selective sweeps in African rice and examined if those selective sweeps showed greater reduction in nucleotide diversity in African rice as compared with its wild relative *O. barthii* and Asian rice and (2) compare the extent of genetic relatedness and population structure of the three rice species using genomewide SNPs.

## Materials and methods

This study was conducted on a total of 3245 accessions and varieties (hereafter referred to as samples) conserved at the AfricaRice genebank (Supplementary Table S1), which represent *O. barthii* (115 samples), African rice (2358 samples) and Asian rice (772 samples). About 92% of the African rice (Ndjiondjop et al. 2017) and 43% of the Asian rice (Ndjiondjop et al. 2018a) samples had been previously used for genetic diversity and population structure studies conducted within each species. Nearly, all samples had also been used for identification of species- and subspecies-diagnostic SNPs for routine genotyping quality control analysis to minimize errors during germplasm collection, acquisition and routine genebank operations (Ndjiondjop et al. 2018b). The detailed procedures for genomic DNA extraction and SNP genotyping using DArTseq™ were described in our previous study (Ndjiondjop et al. 2017). Each sample was genotyped with 31,739 SNPs by DArT Pty Ltd, Australia (http://www.diversityarrays.com) of which 26,073 SNPs (Dataset-1) were physically mapped on to the 12 rice chromosomes and had two alleles each irrespective of their minor allele frequency (Table 1). SNPs that were not physically mapped (5606 SNPs) or SNPs that were physically mapped but were completely monomorphic across all samples (60 SNPs) were excluded from all statistical analyses. Of the 26,073 SNPs in Dataset-1, nearly 89% (23,079 SNPs) were polymorphic across all 3245 samples (Dataset-2), each having a minor allele frequency ranging from 0.01 to 0.496 (Supplementary Table S2).

**Table 1:** Summary of the different datasets, chromosomal distribution and physical map length of SNP markers used in the present study

Chromosome	All SNPs used for genotyping	Dataset-1^a^	Dataset-2^b^	Number of SNPs polymorphic within						Map length (bp)
				*O. glaberrima* (*N* = 2358)	*O. barthii* (*N* = 115)	*O. glaberrima* and *O. barthii* (*N* = 2473)	Lowland *O. sativa* (*N* = 554)	Upland *O. sativa* (*N* = 218)	All *O. sativa* (*N* = 772)	
Chr1	3124	3113	2758	309	570	390	1636	1046	1796	43,229,577
Chr2	2812	2809	2458	281	468	333	1429	742	1566	35,885,080
Chr3	2678	2668	2333	256	508	326	1362	897	1573	36,413,113
Chr4	2378	2368	2077	242	380	300	1243	845	1376	35,497,590
Chr5	1914	1914	1685	212	378	256	916	565	1024	29,763,242
Chr6	2167	2156	1880	232	437	322	1274	848	1361	31,190,621
Chr7	2017	2011	1793	226	399	282	1016	658	1146	29,679,399
Chr8	1877	1877	1668	148	288	188	1298	723	1336	28,429,363
Chr9	1559	1558	1387	157	267	192	849	389	943	22,947,498
Chr10	1576	1573	1453	225	301	253	788	559	878	23,205,328
Chr11	2085	2085	1851	318	449	352	1233	987	1405	29,000,206
Chr12	1946	1941	1736	234	362	290	1062	601	1170	27,504,691
Unmapped	5606									
Total	31,739	26,073	23,079	2840	4807	3484	14,106	8860	15,574	372,745,708
Polymorphism (%)		82.1	88.5	10.9	18.4	13.4	54.1	34.0	59.7	

^a^Dataset-1 refers to the number of SNPs with two alleles irrespective of minor allele frequency (MAF)

^b^Dataset-2 refers to the number of polymorphic SNPs across all 3245 accessions, each with a minor allele frequency of ≥ 0.01

Hapmap input files from TASSEL v.5.2.48 were exported to PHYLIP interleaved format, which were then converted to both MEGA X (Kumar et al. 2016) and ARLEQUIN v.3.5.2.2 (Excoffier and Lischer 2010) formats using PGDSpider (Lischer and Excoffier 2012). We used MEGA X (Kumar et al. 2016) to estimate number of segregating sites, proportion of polymorphic sites (Ps), Theta (*θ*_S_), nucleotide diversity (*θπ*) and Tajima’s *D* test statistic (Tajima 1989). Tajima’s *D* was used to test the null hypothesis of selective neutrality in each species and groups observed within species. To assess the effect of sample size on molecular diversity indices of African rice, we conducted the analyses on (1) a minicore of 350 accessions (Ndjiondjop et al. 2017); (2) subsets of 115 and 163 randomly chosen accessions from the 350 minicore set to get the same sample size as the *O. barthii* used in the current study and that of a recent study (Cubry et al. 2018), respectively, and (3) randomly selected samples of 115 African rice accessions to get the same number of samples as that of *O. barthii* in the present study, which was repeated 20 times. Finally, two additional analyses were also run on two sample sizes of 983 and 1375 African rice accessions proposed based on neighbor-joining cluster and principal component analyses (Supplementary Fig. S1).

**Fig. 1:**
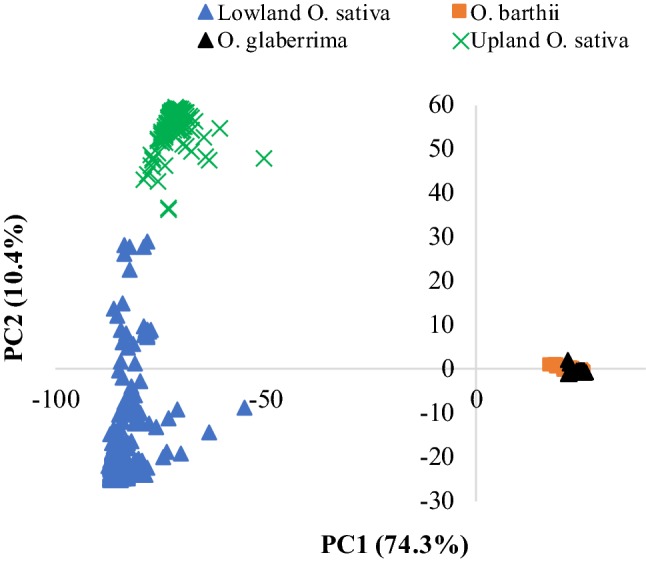
Plot of PC1 and PC2 from principal component analysis of 3245 samples representing *Oryza glaberrima* (2358), *O. barthii* (115) and *O. sativa* (772) using 23,079 polymorphic SNPs. As only 14.4% of the 23,079 SNPs were polymorphic between *O. barthii* and *O. glaberrima,* these two species appeared nearly identical, while *O. sativa* genotypes showed clear separation into upland (218) and lowland (554) ecologies

OmegaPlus v.3.0.2 (Alachiotis et al. 2012) was used to detect selective sweeps that may have undergone positive selection by dividing Dataset-1 into four subsets that corresponded to African rice, *O. barthii*, lowland O*. sativa*, which are primarily indica, and upland *O. sativa*, which are primarily japonica. *O. sativa* samples were divided into lowland and upland ecologies for some statistical analyses due to the distinct population structure observed between these two ecological groups based on results from neighbor-joining cluster analysis (Supplementary Fig. S2) and principal component analysis (Fig. 1). Each subset of data for OmegaPlus was then converted into reference and alternative alleles using the variant call format (VCF) file conversion option in TASSEL v.5.2.48, which refers to the major and minor alleles, respectively. OmegaPlus v.3.0.2 was separately executed on each subset of data as described in a previous study (Alachiotis and Pavlidis 2016), evaluating a grid of 10,000 equidistant physical locations along each subset (Alachiotis and Pavlidis 2018). The average distance between any two consecutive locations was 3105 bp, while each score computation entails the exhaustive evaluation of 1-Mb overlapping windows. The threshold score for declaring selective sweeps to be significant was set as the 99th percentile, so the 1% with the highest scores was retained to represent a candidate selective sweep region. Two or more selective sweeps with an overlapping start and/or end position were considered the same candidate selective sweep. To minimize the length of the manuscript, only peaks (1) that were specific to African rice, (2) had at least three adjacent SNPs within the start and end positions (interval), and (3) had likelihood test scores greater than the minimum threshold value were chosen for further analyses. The start and end physical positions of each selective sweep region were used to search for candidate genes at the Gramene Genome Brower (http://ensembl.gramene.org/genome_browser/index.html).

Additional subsets of genotypic data consisting of all SNPs that mapped across all selective sweeps detected in African rice (Dataset-3), *O. barthii* (Dataset-4), lowland *O. sativa* (Dataset-5) and upland *O. sativa* (Dataset-6) were also used to compute nucleotide diversity in MEGA X (Kumar et al. 2016). Identity-by-state (IBS)-based genetic distance matrix, neighbor-joining cluster analysis, principal component analysis (PCA), analysis of molecular variance (Excoffier et al. 1992) and F_ST_-based pairwise genetic distance matrices (Holsinger and Weir 2009) were conducted by filtering the genotypic data with a minor allele frequency (MAF) of ≥ 0.01 as described in our previous papers (Ndjiondjop et al. 2017, 2018a). The genotypic data used for these analyses include the 23,079 SNPs in Dataset-2 plus other species and group-specific genotypic files created from Dataset-1 by filtering with MAF of ≥ 0.01.

## Results

### Test for selective neutrality and detection of selective sweeps

**Fig. 2:**
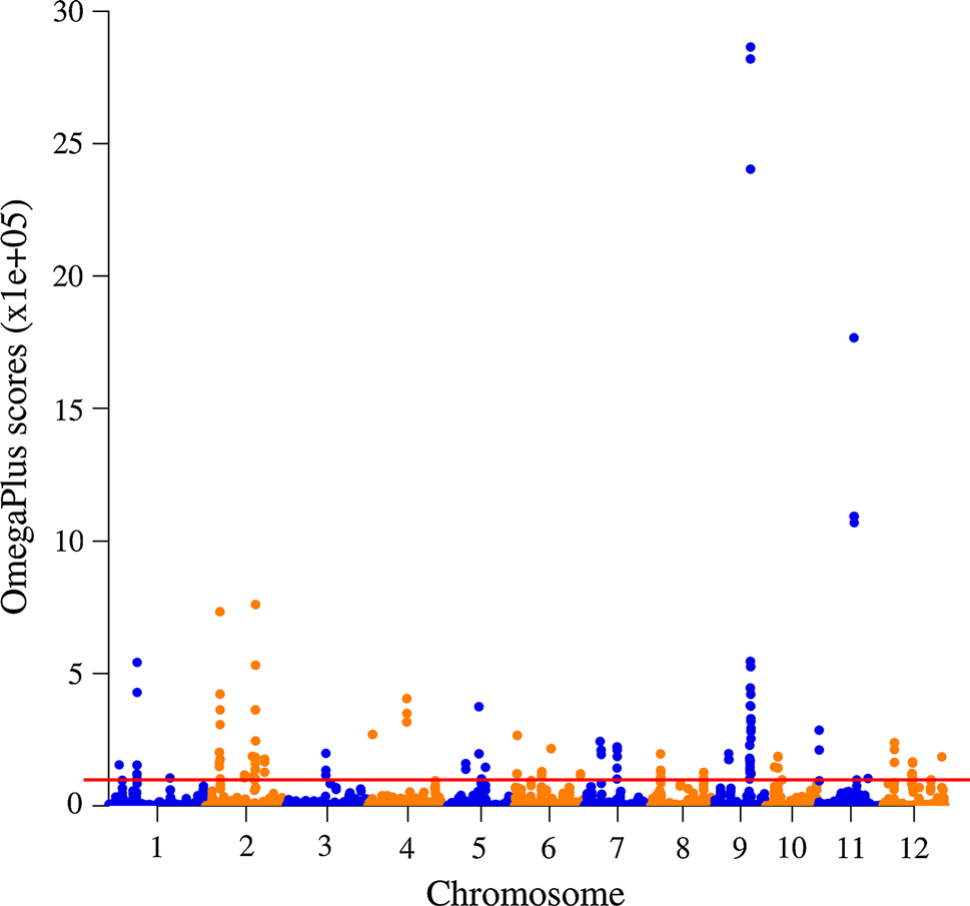
Manhattan plots showing selective sweep regions detected in African rice (*O. glaberrima*). The horizontal solid line indicates the threshold value for declaring candidate selective sweeps (see material and method section for details)

Table 2 summarizes the different evolutionary parameters estimated across the three species. Tajima’s *D* value computed from the 26,073 SNPs in Dataset-1 was positive in the wild *O. barthii* (0.219), but negative in the cultivated African (− 1.381) and Asian rice (− 0.257 for lowland and − 1.513 for upland *O. sativa*). The negative Tajima’s *D* values observed in the two cultivated rice species are indicative of positive selection and reject the null hypothesis that all mutations are selectively neutral. Using OmegaPlus, we identified a total of 37 candidate selective sweep regions in African rice (Fig. 2), 89 regions in lowland Asian rice and 68 regions in upland Asian rice (Table 3, Supplementary Fig. S3). The candidate selective sweeps detected in African rice spanned from 28 to 850 kb and consisted of clusters of markers that varied from 11 to 74 SNPs (Supplementary Table S3); the number of selective sweeps detected per chromosome ranged from 1 on chromosome 3 to 6 on chromosome 2 (Table 3). Overall, there were a total of 780 SNPs that mapped within the 37 candidate selective sweeps detected in African rice (Dataset-3). Only four of the 780 SNPs had MAF ≥ 0.01 in African rice as compared to 88 SNPs in *O. barthii*, 419 SNPs in lowland Asian rice and 275 SNPs in upland Asian rice (Supplementary Table S2). Selective sweeps increase the frequency of beneficial alleles and surrounding variants and may eventually lead to fixation, and recombination and mutation that introduce new alleles are rare (causing alleles of very low frequency), which are evident in Supplementary Table S2.

**Table 2:** Summary of the molecular diversity indices of three rice species based on six datasets of different marker density

Species and dataset^a^	Results from Tajima’s neutrality test^b^					
	*m*	*S*	Ps	*θ*_S_	*θπ*	*D*
All *O. glaberrima*						
Dataset-1	2358	6843	0.262	0.031	0.01620	− 1.381
Dataset-2	2358	6057	0.262	0.031	0.01806	− 1.213
Dataset-3	2358	158	0.203	0.024	0.00083	− 2.641
Dataset-4	2358	320	0.272	0.033	0.01247	− 1.725
Dataset-5	2358	416	0.292	0.035	0.02194	− 1.045
Dataset-6	2358	374	0.263	0.032	0.01391	− 1.565
Group-1 *O. glaberrima*						
Dataset-1	983	4427	0.170	0.023	0.01425	− 1.101
Dataset-2	983	4108	0.178	0.024	0.01592	− 0.979
Dataset-3	983	76	0.097	0.013	0.00070	− 2.598
Dataset-4	983	204	0.174	0.023	0.01378	− 1.169
Dataset-5	983	261	0.183	0.025	0.01866	− 0.690
Dataset-6	983	246	0.173	0.023	0.01423	− 1.115
Group-2 *O. glaberrima*						
Dataset-1	1375	5414	0.208	0.027	0.01499	− 1.270
Dataset-2	1375	4834	0.209	0.027	0.01664	− 1.106
Dataset-3	1375	106	0.136	0.017	0.00089	− 2.612
Dataset-4	1375	244	0.208	0.027	0.00944	− 1.832
Dataset-5	1375	354	0.248	0.032	0.02054	− 1.014
Dataset-6	1375	285	0.201	0.026	0.01176	− 1.546
Minicore *O. glaberrima*						
Dataset-1	350	4664	0.179	0.028	0.01870	− 1.020
Dataset-2	350	4061	0.176	0.027	0.02066	− 0.763
Dataset-3	350	78	0.100	0.016	0.00106	− 2.733
Dataset-4	350	198	0.169	0.026	0.01473	− 1.331
Dataset-5	350	294	0.206	0.032	0.02453	− 0.719
Dataset-6	350	265	0.186	0.029	0.01720	− 1.244
Subset of minicore *O. glaberrima*						
Dataset-1	163	4114	0.158	0.028	0.01948	− 0.986
Dataset-2	163	3632	0.157	0.028	0.02147	− 0.744
Dataset-3	163	54	0.069	0.012	0.00132	− 2.716
Dataset-4	163	179	0.152	0.027	0.01655	− 1.232
Dataset-5	163	265	0.186	0.033	0.02544	− 0.724
Dataset-6	163	231	0.163	0.029	0.01809	− 1.190
Subset of minicore *O. glaberrima*						
Dataset-1	115	3904	0.150	0.028	0.02006	− 0.971
Dataset-2	115	3491	0.151	0.028	0.02211	− 0.751
Dataset-3	115	54	0.069	0.013	0.00162	− 2.756
Dataset-4	115	174	0.148	0.028	0.01790	− 1.180
Dataset-5	115	244	0.171	0.032	0.02622	− 0.615
Dataset-6	115	218	0.153	0.029	0.01872	− 1.164
*O. barthii*						
Dataset-1	115	5685	0.218	0.041	0.04366	0.219
Dataset-2	115	4919	0.213	0.040	0.04396	0.326
Dataset-3	115	108	0.138	0.026	0.02020	− 0.731
Dataset-4	115	254	0.216	0.041	0.02258	− 1.478
Dataset-5	115	370	0.259	0.049	0.05148	0.184
Dataset-6	115	317	0.223	0.042	0.04903	0.563
*O. sativa* spp. indica (lowland)						
Dataset-1	554	19,090	0.732	0.106	0.09722	− 0.257
Dataset-2	554	17,327	0.751	0.109	0.10654	− 0.066
Dataset-3	554	585	0.750	0.109	0.08782	− 0.581
Dataset-4	554	819	0.697	0.101	0.09336	− 0.232
Dataset-5	554	856	0.600	0.087	0.06371	− 0.810
Dataset-6	554	1015	0.714	0.104	0.09803	− 0.163
*O. sativa* spp. japonica (upland)						
Dataset-1	218	12,734	0.488	0.082	0.04340	− 1.513
Dataset-2	218	12,066	0.523	0.088	0.04751	− 1.474
Dataset-3	218	398	0.510	0.086	0.04041	− 1.679
Dataset-4	218	607	0.517	0.087	0.04355	− 1.588
Dataset-5	218	604	0.424	0.071	0.04088	− 1.356
Dataset-6	218	426	0.299	0.050	0.01159	− 2.447
Both lowland and upland *O. sativa*						
Dataset-1	772	20,642	0.792	0.110	0.17332	− 5.807
Dataset-2	772	18,763	0.813	0.113	0.19298	− 6.165
Dataset-3	772	654	0.838	0.116	0.18026	1.639
Dataset-4	772	910	0.774	0.107	0.17936	1.999
Dataset-5	772	961	0.674	0.093	0.13485	1.324
Dataset-6	772	1067	0.751	0.104	0.15331	1.413

^a^Number of sequences or sample size (*m*); number of segregating sites (*S*); proportion of polymorphic sites (Ps) = *S*/*n*); *θ*_S_ = Ps/a1; nucleotide diversity (*θπ*); Tajima test (*D*)

^b^Dataset-1 = 26,073 SNPs; Dataset-2 = 23,079 SNPs; Dataset-2 = 780 SNPs; Dataset-4 = 1175 SNPs; Dataset-5 = 1426 SNPs; Dataset-6 = 1421 SNPs. See Table 1 for details

**Table 3:** Chromosomal distribution of number of selective sweep regions identified in three rice species

Chromosome	*O. glaberrima*	Lowland *O. sativa*	Upland *O. sativa*	*O. barthii*
1	3	11	8	6
2	6	11	6	1
3	1	15	5	2
4	2	9	10	3
5	4	5	8	3
6	5	10	4	2
7	3	8	4	1
8	2	2	3	1
9	2	4	10	2
10	2	3	5	4
11	3	5	3	6
12	4	6	2	5
Grand total	37	89	68	36

Selective sweeps were more common in Asian rice than in African rice (Table 3). Each selective sweep identified in lowland and upland Asian rice spanned from 55 to 771 kb and from 47 to 1045 kb, respectively, and harbored clusters of SNPs ranging from 6 to 54 and from 10 to 76 markers (Supplementary Table S3). Five of the 89 selective sweeps identified in lowland Asian rice (3: 17,580,209–17,811,736, 5: 15,191,088–15,198,145, 7: 7,420,481–7,526,572, 9: 15,703,948–16,396,541 and 11: 16,030,588–16,164,430) and seven of the selective sweeps in upland Asian rice (1: 4,537,132–4,724,160, 2: 6,069,007–6,374,310, 2: 21,994,604–22,316,127, 8: 23,755,429–23,755,429, 9: 15,547,000–15,556,789, 11: 442,387–442,387 and 11: 22,278,851–22,445,600) were partial overlapping with selective sweeps detected in African rice (Supplementary Tables S2, S3). Although the positive Tajima’s *D* value in *O. barthii* supports the null hypothesis of selective neutrality, results of OmegaPlus revealed 36 selective sweeps in this wild species of which six regions (3: 17,716,633–17,811,736, 5: 13,940,602–14,087,325, 9: 16,021,480–16,396,541, 10: 3,705,613–3,806,404, 11: 442,387–595,142 and 11: 22,228,554–22,267,775) were shared with those detected in African rice (Supplementary Tables S2, S3). Overall, we identified a total 1353 SNPs, 1426 SNPs and 1421 SNPs that fell within the selective sweeps detected in *O. barthii*, lowland and upland Asian rice, respectively, which were used for computing molecular diversity indices (see below).

### Candidate genes in selective sweeps detected in African rice

To gain insight into possible roles of each selective sweep in African rice, we compiled a list of 901 protein coding candidate genes found within the 37 selected regions (Supplementary Table S4). There were two to ninety-two genes per selective sweep region of which some had known functions, descriptions of which can be found in Supplementary Table S3. As shown in the Manhattan plot in Fig. 2, the highest test scores (1.0 × 10^5^–28.6 × 10^5^) were observed in 850 kb interval on chromosome 9 (9: 15,547,000–16,396,541). This region harbored clusters of 87 protein coding genes (Supplementary Table S4), including DEEPER ROOTING 1 (DRO1), that play role for adapting to the dry sub-Saharan region and DELAYED SEED GERMINATION 1 (OsDSG1) that prevent germination when there is insufficient moisture in the soil and under storage conditions. The role of other candidate genes with known function has been summarized in Supplementary Table S3 and described in detail in the discussion section.

### Molecular diversity indices

Of the 26,073 SNPs in Dataset-1, the number of segregating sites (6843) and proportion of polymorphic sites (0.262) in African rice were nearly 20% greater than those of *O. barthii,* but 64.2% and 46.3% smaller than those of the lowland and upland Asian rice, respectively (Table 2). Theta (ϴ) in African rice was 0.031, which is 76.7% of that of *O. barthii* (0.041), 29.6% of the lowland and 38.4% of the upland Asian rice. Nucleotide diversity (*π*) in African rice was 0.016, which accounted for 37.1%, 16.7% and 37.3% of the values observed in *O. barthii* (0.044), lowland (0.097) and upland (0.043) Asian rice, respectively. The number of segregating sites, proportion of polymorphic sites and ϴ and π values computed from the 23,079 SNPs in Dataset-2 that were polymorphic across all 3245 samples at a MAF ≥ 0.01 were similar to that of Dataset-1.

Using genotype data of the 780 SNPs (Dataset-3) that fell within the 37 selective sweeps detected in African rice, we compared the changes in nucleotide diversity across the three species. Nucleotide diversity in African rice was 8.3 × 10^−4^, which accounted for just 4.1% of that of *O. barthii*, 0.9% and 2.1% of the lowland and upland Asian rice, respectively (Table 2). On the other hand, nucleotide diversity of the African rice computed from SNPs that fell within the selective sweeps identified in *O. barthii* (Dataset-4) and lowland Asian rice (Dataset-5) accounted for 55.2% and 34.4% of that of *O. barthii* and lowland Asian rice, respectively, which are even higher than the values obtained from the entire 26,073 SNPs in Dataset-1. These results demonstrate a more severe reduction in genetic variation in African rice due to selective sweeps detected in this species than the selective sweeps detected in the other two species. The nucleotide diversity of African rice as calculated from SNPs in selective sweeps identified in upland Asian rice (Dataset-6) was sevenfold greater than the value obtained in Dataset-3, but smaller than that of Dataset-1. The latter is not surprising due to not only much smaller Tajima *D* value observed in upland than the lowland Asian rice (Table 2), but also the presence of more common selective sweeps between the African rice and the upland Asian rice (Supplementary Table S2). Both African rice and *O. barthii* showed reduction in nucleotide diversity in Dataset-3 than Dataset-1, but the reduction in the former was 19-fold (*π* = 1.6 × 10^−2^ in Dataset-1 vs. 8.3 × 10^−4^ in Dataset-3) as compared to just twofold in *O. barthii* (*π* = 4.4 × 10^−2^ in Dataset-1 vs. 2.0 × 10^−2^ in Dataset-3). On the contrary, the changes in nucleotide diversity in lowland and upland Asian rice were very minimal irrespective of the datasets; the only exceptions were Dataset-5 in the lowland Asian rice and Dataset-6 in the upland Asian rice that showed about from two to threefold smaller nucleotide diversity values as compared to the whole 26,073 SNPs.

To assess the effect of sample size on the molecular diversity indices in African rice, we repeated the analyses on five additional samples sizes (*N* = 115, 163, 350, 983 and 1375) using the 26,073 SNPs. Nucleotide diversity of African rice computed from these five smaller sample sizes accounted from 30.5 to 45.9% of that of *O. barthii*, from 13.5 to 20.6% of that of lowland Asian rice and from 30.1 to 46.2% of that of upland Asian rice (Table 2, Supplementary Table S5). Overall, nucleotide diversity values estimated from smaller sample sizes showed an increase or decrease up to 8.9% as compared to the entire 2358 accessions; hence, the severe reduction in nucleotide diversity observed in African rice than both its wild progenitor and Asian rice was very consistent irrespective of sample size (Supplementary Fig. S4) and marker density.

### Genetic relatedness and population structure

Genetic distance between pairs of all 3245 samples computed from the 23,079 SNPs that were polymorphic with a MAF ≥ 0.01 in Dataset-2 varied from 0.001 to 0.662, with an overall average of 0.243 (Supplementary Table S6). Nearly 56% of the pairs of samples differed by just < 5% of the scored alleles. All pairs of African rice samples, 50.2% of the *O. barthii* pairs and 5.2% of Asian rice pairs differed by < 5% of the alleles. Approximately 36% of the pairs of samples differed by more than 50% of the alleles in Dataset-2 which was due to greater distance among pairs of samples belong to different species. Neighbor-joining tree constructed from the genetic distance matrix of all 3245 samples showed three major groups (Supplementary Fig. S2). The first group consisted of all samples belonging to the African rice and *O. barthii.* The second and third groups consisted of all Asian rice samples adapted to the lowland (which are primarily indica) and the upland (primarily japonica) ecologies in Africa, as summarized in Supplementary Table S1. The first two principal components from PCA performed across all three species accounted for 84.7% of the molecular variation observed across the 3245 samples. A plot of PC1 (74.3%) and PC2 (10.4%) showed clear population structure in the same way as the neighbor-joining analysis (Fig. 1).

**Fig. 3:**
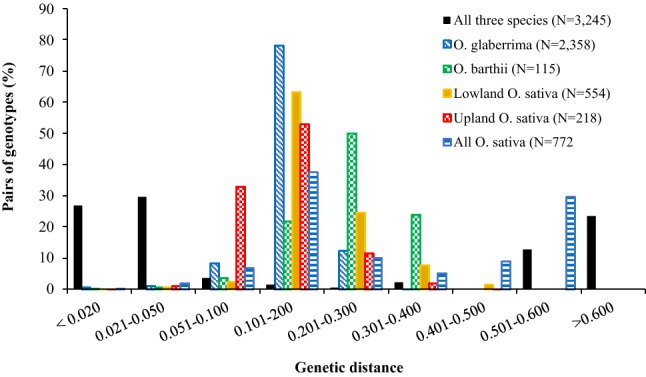
Genetic distance summaries between pairs of 3245 samples computed from SNPs that were polymorphic across all three species (23,079 SNPs), *O. glaberrima* (2840 SNPs), *O. barthii* (4807 SNPs), lowland *O. sativa* (14,106 SNPs) and upland *O. sativa* (8860 SNPs). The number of samples is shown in the legend within brackets

We then performed separate analyses on genotypic data of each species to get a better insight into the extent of genetic variation among accessions belonging to each species. Among the 26,073 SNPs in Dataset-1, the number of SNPs that were polymorphic within each species (at a MAF ≥ 0.01) was 10.9% (2840 SNPs) in African rice, 18.4% (4807 SNPs) in *O. barthii*, 54.1% in lowland Asian rice and 34.0% in upland Asian rice (Table 1). The proportion of polymorphism in African rice accounted for 59.1%, 20.1% and 32.1% of the polymorphism observed within *O. barthii,* lowland and upland Asian rice, respectively. Genetic distance estimated from SNPs that were polymorphic within each species/group varied from 0.004 to 0.308 in African rice, from 0.011 to 0.349 in *O. barthii*, from 0.013 to 0.466 in lowland Asian rice and from 0.018 to 0.416 in upland Asian rice (Supplementary Table S7). In African rice, the genetic distance values for about 78% and 12% of the pairs of accessions varied from 0.101 to 0.200 and from 0.201 to 0.300, respectively, with none of the pairs having a genetic distance exceeding 0.308. On the contrary, approximately 22%, 50% and 24% of the pairs of *O. barthii* accessions had genetic distance values ranging from 0.101 to 0.200, from 0.201 to 0.300 and from 0.301 to 0.400, respectively (Fig. 3, Supplementary Table S7).

The overall average distance observed within African rice (0.152) accounted for 63.1% and 80.0% of the average distance observed in *O. barthii* (0.241) and lowland Asian rice (0.190), respectively, but it was 12.6% greater than that of upland Asian rice (0.135) (Supplementary Table S7). The phylogenetic tree constructed from the genetic distance matrix computed from 3484 SNPs that were polymorphic in both African rice and *O. barthii* showed three major groups, with the African rice samples partitioned into six subgroups (Supplementary Fig. S5). Plots of PC1 and PC2 from PCA (Supplementary Fig. S6**)** showed similar pattern as the cluster analysis. Detailed results of 2179 of the 2358 (94.4%) African rice samples have been presented in our previous study (Ndjiondjop et al. 2017). The addition of 179 African rice samples and the inclusion of the *O. barthii* samples changed the overall results only a little. The partitioning of the overall molecular variance (AMOVA) into four hierarchical levels corresponding to African rice, *O. barthii*, lowland Asian rice and upland Asian rice revealed that differences among groups accounted for 92.4% of the total variation (Table 4). Pairwise *F*_ST_ computed for African rice against *O. barthii*, lowland Asian rice and upland Asian rice was 0.276, 0.939 and 0.964, respectively. *F*_ST_ values between *O. barthii* vs. lowland, *O. barthii* vs. upland and lowland vs upland Asian rice were 0.844, 0.919 and 0.0.7389, respectively.

**Table 4:** Analysis of molecular variance (AMOVA) for the extraction of SNP variation among and within groups (populations)

Category	No. of SNPs used for analysis	Source of variation	*d.f*.	Sum of squares	Variance components	Percentage of variation
Four groups (*O. glaberrima*, *O. barthii*, lowland *O. sativa* and upland *O. sativa*)	23,079	Among groups	3	7,600,354.2	5358.1	92.8
		Within groups	3241	1,347,725.3	415.8	7.2
		Total	3244	8,948,079.4	5774.0	100.0
Two groups (*O. glaberrima* and *O. barthii*)	23,079	Among groups	1.0	18,837.7	84.9	27.65
		Within groups	2471.0	548,912.1	222.1	72.35
		Total	2472	567,749.8	307.0	100.0
Two groups (*O. glaberrima* and *O. barthii*)	3484	Among groups	1.0	18,466.0	83.2	27.67
		Within groups	2471.0	537,451.2	217.5	72.33
		Total	2472	555,917.2	300.7	100.0

## Discussion

### Genetic relatedness, sample sizes and misclassification

Details comparing genetic distance, relatedness and population structure within African rice and Asian rice have already been presented in our previous studies (Ndjiondjop et al. 2017, 2018a). The primary focus of the present study was to compare marker polymorphism, nucleotide diversity, genetic relatedness and population structure among the three AA-genome rice species using DArTseq-based genomewide SNPs and SNPs that fell within selective sweeps identified in African rice. Polymorphism levels (Table 1), nucleotide diversity (Table 2) and genetic distances (Supplementary Table S7) in African rice were all greatly reduced compared to *O. barthii*, which are consistent with previous studies irrespective of marker types and density (Joshi et al. 2000; Ishii et al. 2001; Park et al. 2003; Semon et al. 2005; Kwon et al. 2006; Li et al. 2011; Orjuela et al. 2014; Wang et al. 2014; Meyer et al. 2016; Ndjiondjop et al. 2017). The lower genetic distance values among most pairs of African rice samples were also evident in both phylogenetic tree (Supplementary Fig. S2) and PCA plot (Fig. 1); these plots of genetic structure and relationships also agree with previous studies (Li et al. 2011; Orjuela et al. 2014; Cubry et al. 2018). Orjuela et al. (2014) found two subgroups for African rice and three subgroups for *O. barthii* primarily based on geography and that African rice and *O. barthii* were closer together than either of them compared to Asian rice. Wang et al. (2014) found five groups and that all *O. barthii* and African rice accessions from Gambia, Guinea, Senegal and Sierra Leone formed two admixture groups (OB-V and OB-IV), whereas the other accessions sampled outside the domestication center formed three additional groups. In the current study, we found six groups of African rice and that the group membership of the *O. barthii* samples generally agrees with their country of origin but not ecology. However, nearly 40% of the *O. barthii* accessions were clustered together with African rice accessions, which differed between the current study and other studies (Orjuela et al. 2014; Wang et al. 2014), which may be due to differences in the genetic background of the germplasm and sample size (Iwamoto et al. 1999; Lu et al. 2000; Nagano et al. 2000; Ishii et al. 2001; Park et al. 2003; Ren et al. 2003; Zhu and Ge 2005; Duan et al. 2007; Zhang et al. 2014; Wambugu et al. 2015; Yin et al. 2016).

The close relationship between *O. barthii* and many of the African rice accessions undoubtedly reflects the direct relationship between the two species, but it may also be caused by some misclassification of accessions. In previous studies, we found out that 3.1% of accessions across four rice species were misclassified/misidentified (Ndjiondjop et al. 2018b), which was smaller than the 4–21% misclassification reported in other studies (Buso et al. 2001; Girma et al. 2012; Orjuela et al. 2014; Mason et al. 2015). To minimize errors due to misclassification during germplasm collection, acquisitions and routine genebank operations, our group identified 332 species- and subspecies-specific diagnostic SNP markers in *O. glaberrima/O. barthii*, *O. sativa* spp. indica, *O. sativa* spp. japonica and *O. longistaminata* that can be used for genotyping quality control analysis; however, none of the markers were diagnostic between *O. glaberrima* and *O. barthii* (Ndjiondjop et al. 2018b), which is another indicator of very close genetic relationship between these two species.

We used varying sample sizes, varying marker numbers and three methods to detect genomic regions with an evidence of selective signature during domestication: (1) the identification of selective sweeps, (2) the genetic diversity ratio of the wild and cultivated species and (3) the identification of loci that had undergone extreme genetic differentiation (Cubry et al. 2018), which all revealed consistently much smaller genetic variation in African rice than its wild progenitor *O. barthii* and Asian rice, irrespective of sample size and marker density. The reduction in genetic diversity in African rice as compared to its wild progenitor was consistent with previous studies conducted on smaller number of samples ranging from 19 to 163 accessions, and with molecular markers or whole genome sequencing (Li et al. 2011; Wang et al. 2014; Meyer et al. 2016; Win et al. 2017; Cubry et al. 2018).

### Selective sweeps in African rice

A severe reduction in nucleotide diversity was observed across diverse sample size of African rice when 780 SNPs in Dataset-3 that mapped within the 37 candidate selective sweep regions detected in this species were used for analysis compared to those detected in *O. barthii* and Asian rice (Table 2). Such sharp reduction in nucleotide diversity in African rice in the selective sweeps regions is likely due to positive selection during and/or after domestication as compared with the other two species, as has been reported around known rice domestication genes (Li et al. 2006, 2011; Olsen et al. 2006; Jin et al. 2008; Zhang et al. 2009; He et al. 2011; Zhu et al. 2011; Huang et al. 2012; Hua et al. 2015; Civáň and Brown 2017; Win et al. 2017; Lv et al. 2018). Some of the most widely cited domestication genes reported in African and Asian rice include the semidwarf gene (SD1) (Cho et al. 1994) and a major effect QTL for grain shattering (qSH1) that accounted for 36% of yield difference between indica and japonica cultivars (Konishi et al. 2006; Onishi et al. 2007) on chromosome 1; long kernel 3 (LK3) or grain size 3 (GS3) (Fan et al. 2006; Takano-Kai et al. 2009) on chromosome 3; SHAT1, which encodes APETALA2 (AP2) transcription factor (Zhou et al. 2012), shattering 3 (Sh3), shattering 4 (Sh4) (Inoue et al. 2015; Wu et al. 2017), hull color (Bh4) (Zhu et al. 2011; Vigueira et al. 2013) and LONG AND BARBED AWN1 (LABA1) (Hua et al. 2015) genes on chromosome 4; SHATTERING 5 (SH5) and a major effect grain width QTL (qSW5/GW5) on chromosome 5; SHATTERING-H, Red grain color (Rc) and Prostrate growth 1 (PROG1), which controls the transition from prostate to erect growth habit (Tan et al. 2008) on chromosome 7.

Although we were not confidently able to locate the same domestication genes in our study, we found various candidate genes with known functions that mapped within the 37 selective sweep regions detected in African rice (Supplementary Tables S3, S4). Some of the candidate genes that fell within the selective sweep region on chromosome 9 with the highest Omega scores were a Jasmonate ZIM-domain protein that induces resistance to bacterial blight (Yamada et al. 2012); DEEPER ROOTING 1 (DRO1) that controls root system architecture and drought avoidance and increases grain yield under drought conditions (Uga et al. 2013); ALDEHYDE DEHYDROGENASE 7, which is required for seed maturation and maintenance of viability during storage (Shin et al. 2009); DELAYED SEED GERMINATION 1 (OsDSG1) that controls seed germination in storage and stress responses in rice (Park et al. 2010); ETHYLENE RESPONSE FACTOR 72, which regulates expression of a wide variety of downstream target genes related to stress response and development (Phukan et al. 2017) and CELLULOSE SYNTHASE-LIKE C2 protein, which is required for cellulose synthesis and larger growth (Gu and Somerville 2010). Other genes of known function that fell within the second selective sweep on chromosome 9 (9: 6,267,139–6,508,307 bp) are SUBMERGENCE 1B (Sub1B) and Sub1C, which are involved in rice tolerance to submergence (Fukao et al. 2006), and NA+/H+ANTIPORTER 5, which is a sodium/hydrogen exchanger subfamily protein that enhances salinity tolerance in rice (Verma et al. 2007; Khan 2011), both of which are needed when rice is grown under flood irrigation.

The second highest Omega scores were observed for a selective sweep region identified on chromosome 11, which spans 153 kb (11: 16,030,588–16,183,929 bp) and harbors 5 candidate genes (Supplementary Tables S3, S4). The latter includes an aminotransferase-like enzyme, which is involved in a number of metabolic activities, including abiotic stress in rice (Kothari et al. 2016); a Bx2-like protein that is associated with iron toxicity in rice (Finatto et al. 2015) and protein kinase domain containing protein that catalyze the transfer of the phosphate from nucleotide triphosphates to one or more amino acid residues in a protein substrate side chain. Of the candidate genes that mapped within the 695 kb interval on chromosome 2 (2: 5,973,055–6,668,406), PLASTIDIC NUCLEOTIDE TRANSPORT PROTEIN is involved in the carbon flow related to starch metabolism and thus larger, more nutritious grain (Toyota et al. 2006); RECEPTOR-LIKE CYTOPLASMIC KINASE plays a role in plant signaling (Liang and Zhou 2018); PLANT DEFENSIN 1.2 is involved in defense against fungi (Silverstein et al. 2005); CLASS-1-TYPE HISTONE DEACETYLASE is involved in reproductive development, seed morphology and plant architecture (Jang et al. 2003); gamma ray-induced Leucine-rich repeat receptor-like kinase (LRR-RLK) plays key roles in abiotic stresses tolerance (Park et al. 2014); ALLENE OXIDE SYNTHASE 3 plays a role in the biosynthesis of jasmonic acid, while β-galactosidase is involved in plant defense and the metabolism of galactose-rich polymers (Esteban et al. 2003).

## Conclusion

The present study was conducted on a large collection of genotypes representing the three AA-genome *Oryza* species genotyped using DArTseq-based genotyping by sequencing. Our study clearly demonstrated a narrowing of genetic diversity within the cultivated African rice as compared with its wild progenitor. The reduction in the overall nucleotide diversity in African rice was 14–20-fold (depending on sample size) when the analyses were conducted on the genotype data of 780 SNPs that fell within 37 selective sweep regions identified in this species. These regions contained various annotated genes other than the well-known domestication genes whose functions provide additional clues as to how domestication proceeded from *O. barthii* to cultivated African rice.

### Author contribution statement

MNN conceived, designed and supervised the experiments, secured funding and partly drafted the paper; AG and SBK were responsible for sample preparation, DNA extraction and/or compilation of passport information; NA conducted the selective sweep analyses; KS conducted most analyses and wrote most part of the paper; DZ and PP contributed to and edited the paper. All authors read and approved the paper.

## Electronic supplementary material

Below is the link to the electronic supplementary material.

Supplementary material 1 (7z 9 kb)
